# Does intraspecific competition cause oxidative stress? Influence of biotic and abiotic factors on antioxidant system of an invasive round goby

**DOI:** 10.1002/ece3.10795

**Published:** 2023-12-21

**Authors:** Dagmara Błońska, Ali Serhan Tarkan, Bartosz Janic, Mariusz Tszydel, Bożena Bukowska

**Affiliations:** ^1^ Department of Ecology and Vertebrate Zoology, Faculty of Biology and Environmental Protection University of Lodz Łódź Poland; ^2^ Department of Basic Sciences, Faculty of Fisheries Muğla Sıtkı Koçman University Muğla Turkey; ^3^ Department of Biophysics of Environmental Pollution, Faculty of Biology and Environmental Protection University of Lodz Łódź Poland

**Keywords:** antioxidant defence, intraspecific interactions, invasive species, *Neogobius melanostomus*, oxidative damage

## Abstract

Changes in oxidative status represent organismal response to stressful external stimuli. While there is substantial knowledge on the influence of abiotic factors on the antioxidant system of different organisms, the impact of biotic factors remains largely unexplored. The aim of the present study was to evaluate the effect of acute competitive interactions on oxidative stress. Territory‐resident and intruder round goby *Neogobius melanostomus* individuals were experimentally subjected to competition for limited shelter resource in three treatments (lasting 1, 6 and 12 h), and oxidative stress parameters (total antioxidant capacity, catalase activity, reduced glutathione, lipid peroxidation), as well as behaviour (time spent in the shelter, guarding the shelter and aggression) were measured. All tested biochemical parameters reached higher values in the liver than in the muscle tissue. Fish behaviour and antioxidant defence did not show any potential relationships reflecting changes in antioxidant status and aggression. Particularly, there was no difference between resident and intruder fish in oxidative stress parameters. We compared our results to the outcome of our previous studies (similar experimental protocol and species) but with acute heat shock as a stressor instead of competition. The higher temperature was found to be a stronger stressor than the competition, most pronounced in total antioxidant capacity and oxidative damage.

## Graphical abstract

We verified the effect of biotic stressor—acute competition regarding oxidative stress parameters in round goby and compared it to abiotic stressor (elevated temperature). Our results demonstrated that acute heat shock elicit a higher response of antioxidant defence than intraspecific interactions.

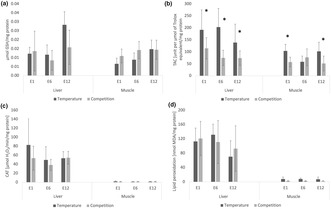

## 1 INTRODUCTION

A general feature of all living cells is their vulnerability to external stimuli, including environmental stressors. Stress‐induced stimuli develop a universal response in organisms independent of the affecting factor. One of the reactions to a number of anthropogenic and natural stressors is the enhancement of steady‐state reactive oxygen species (ROS) concentration called ‘oxidative stress’ (Birnie‐Gauvin et al., 2017; Lushchak, 2011; Monaghan et al., 2009). ROS are naturally generated as a side effect of aerobic metabolism and are usually counterbalanced by antioxidant system consisting of low (e.g. reduced glutathione, ascorbic acid—Vit C, carotenoids) and high (e.g. enzymes as catalase, peroxidases and metallothioneins) molecular mass antioxidants (Blier, 2014; Hermes‐Lima, 2004; Lushchak, 2011). Consequences of prolonged ROS disturbance include changes in cellular metabolism and regulation with resulting damage to cellular constituents (Sopinka et al., 2016). If the system recovers relatively quickly (minutes to hours) from deleterious effects of ROS, the stress is called acute, while longer rehabilitation evokes chronic oxidative stress (Lushchak, 2011, 2014). Efficient elimination of ROS by antioxidant system enables the system to return to its initial state. The elicitation of oxidative stress is most often registered by changes in the level of scavengers (e.g. low molecular mass antioxidants) or antioxidant enzymes activity, and/or oxidised cellular constituents (Lushchak, 2011, 2014).

Oxidative stress development is frequent response to considerable stressors (Lushchak, 2011; Sopinka et al., 2016). Organisms are exposed to various environmental stressors, including both abiotic factors such as changes in habitat structure or pollution, and biotic factors such as inter‐ and intra‐specific interactions. Despite the fact that oxidative stress may develop in response to any significant stressor, most studies have focused on the influence of abiotic factors such as heavy metals, xenobiotics, pesticides or physical factors such as temperature or salinity (Birnie‐Gauvin et al., 2017; Lushchak, 2011). Biotic factors such as competition or predation have received much less attention but have started to attract more interest in the last decade (Janssens & Stoks, 2013; Jermacz, Kletkiewicz, Nowakowska, et al., 2020; Li et al., 2016; Paul et al., 2021).

Aquatic animals possess similar mechanisms to response to oxidative stress as other groups, and they are also suggested to be better and competitive models for such studies (Blier, 2014; Lushchak, 2011). The synergistic effect of predator and thermal stressors examined by Jermacz, Kletkiewicz, Krzyzynska, et al. (2020) indicated increased cellular damage under predation risk in various thermal regimes and reduced activity of antioxidant system in amphipods. Paul et al. (2021) showed inconsistent results on fish *Gobius paganellus*, whose antioxidant response was not induced due to exposition to predator pressure and elevated temperature; however, chronic exposure to predation risk revealed significant DNA damage. Non‐consumptive effect of predator–prey relationship alone can also induce oxidative damage (Janssens & Stoks, 2013; Jermacz, Nowakowska, Kletkiewicz, & Kobak, 2020). However, prolonged and constant contact with predation cues may result in habituation (Jermacz, Nowakowska, Kletkiewicz, & Kobak, 2020) and even decrease the level of damage (Jermacz, Kletkiewicz, Nowakowska, et al., 2020). At the same time, antioxidant defence measured via various biomarkers in response to the predation risk decreased (Janssens & Stoks, 2013; Jermacz, Kletkiewicz, Nowakowska, et al., 2020; Slos & Stoks, 2008). Notably, higher or lower antioxidant status does not directly translate into oxidative damage but depends on upregulation of antioxidant defence as well as ROS concentration (Monaghan et al., 2009).

The predator–prey relationship is perceived particularly intensively by prey, and this interaction is asymmetric. However, organisms at all trophic levels must regularly compete for various resources, whether intra‐ or interspecifically. Competition is usually evaluated at the organismal level, with changes in behaviour (e.g., aggression, fighting) (Dubs & Corkum, 1996; Englund & Olsson, 1990; Grabowska et al., 2016). This type of interaction has been poorly studied in terms of its effect on oxidative stress, with only few studies investigating this subject (Border et al., 2019; Mentesana & Adreani, 2021; Sureda et al., 2017). Dijkstra et al. (2015) tested whether three female morphs of cichlid *Neochromis omnicaeruleus* showed differences in oxidative stress parameters with regard to their various territorial and aggressive behaviours, revealing no significant differences. Similar results, with no difference in either oxidative damage or antioxidant capacity, were obtained for two male morphs of other cichlid species *Astatotilapia burtoni*, but one morph was more sensitive than the other concerning competition (Border et al., 2019). Experiments conducted separately on native and alien bivalves suggested potential competitive advantage regarding oxidative stress in favour of the latter (Oliveira et al., 2015). Quite recently, a few articles focusing on the influence of social status and dominance on oxidative stress parameters in cichlid fish were published (Border et al., 2019, 2021; Funnell et al., 2021). Competition for access to resources connected with social dominance was shown to influence the balance between ROS and antioxidants. Although it is not straightforward whether subordinates or high‐ranking individuals experience similar intensities of oxidative stress, such interactions remain substantial for organisms (Funnell et al., 2021). Studies of Funnell et al. (2021) confirmed that social dominance has its oxidative costs with regard to reproduction. Comparison of female and male of cichlid species *A. burtoni* revealed differences between the sexes and showed that increased oxidative stress was connected with increased investment in reproduction, which was pronounced in males (Funnell et al., 2021). Another kind of competitive interactions is sibling competition, mostly studied in birds, indicating associations between oxidative stress intensity and brood size (Costantini, 2008; Costantini et al., 2010). However, research regarding the potential of competitive interactions to induce oxidative stress remains poorly explored.

The aim of the present study was to evaluate whether direct acute competition can influence the antioxidant status and oxidative damage in the round goby *Neogobius melanostomus*, one of the most invasive aquatic species worldwide (Kornis et al., 2012). The round goby has rapidly expanded throughout European water bodies and the Laurentian Great Lakes (Kornis et al., 2012; Roche et al., 2013) and is known to have detrimental effects on ecosystems due to its efficient competition with native species for food resources and shelter (Dubs & Corkum, 1996; Kessel et al., 2011; Kornis et al., 2012). Under experimental conditions, we evaluated the effect of competition for a shelter between two males of round gobies during the spawning season, when males protect the eggs laid in the nest and aggressively defend it (Kornis et al., 2012; Meunier et al., 2009). Competitive interactions are therefore most intense during this crucial period. We used a well‐established experimental protocol (Dubs & Corkum, 1996; Grabowska et al., 2016; Kakareko et al., 2013; Kessel et al., 2011) and measured several oxidative stress parameters (catalase activity, reduced glutathione, total antioxidant capacity and lipid peroxidation) in the competing males. Additionally, to estimate the strength of competition as a stressor for round goby, we compared our results with our previous study in which we exposed round goby to acute heat shock, a known intense and strong stressor, under similar laboratory conditions (Błońska et al., 2021).

## 2 METHODS

### 2.1 Fish

Specimens of round goby were collected on 6th May 2019 in the Radunia River in Pruszcz Gdański (permission obtained from water tenant—Polish Angling Association in Gdańsk L.Dz.611/19), Poland (54°16′50″ N, 18°38′22″ E). Only males were collected in this study due to their increased aggressiveness and territoriality during spawning, which was fundamental for the aim of the study. Mature males, ranged 95–160 mm in total length (mean 124), were easy do determine during reproductive period based on the shape of urogenital papillae (Charlebois et al., 1997). Fish were transported in aerated tanks to the laboratory, where they were acclimated for 24 h, and then placed in 70 L aquaria (4–5 individuals) equipped with halves of PVC pipes to provide shelter (5 cm long, exceeding the number of fish to avoid competition). Fish were marked by randomly cutting part of their pectoral fin: right—residents and left—intruders, and each status type was grouped in the same aquaria. This procedure enabled to distinguish between individuals after the experiment and on recorded files. During the acclimation (including the time after removing part of the pectoral fin), we recorded no mortality. All stocking aquaria were connected in a flow‐through system (the same volume exchanged constantly) to maintain standard conditions. The photoperiod was set 14 h:10 h (day:night) to mimic natural conditions, water temperature maintained at 18–19°C and tanks were constantly mildly aerated. Fish were fed every second day with frozen chiromonid larvae. The round goby individuals were kept in such conditions for 3 weeks.

### 2.2 Experimental protocol

Experiments were conducted in 45 L non‐transparent, mildly aerated tanks equipped with a single shelter (similar to those used in the stocking aquaria). The fish were not fed before (last feeding a day before the experiment) and during the experiment. Each resident individual was kept for 24 h separately in the tank to acclimate to the experimental conditions and establish territory and shelter. After 24 h, to each experimental tank one intruder (another individual) was added. Before introduction to the tank, every specimen was measured, and fish were matched with similar sizes (no significant difference between residents and intruders; *t*_34_ = 0.7469, *p* = .4603). In the present study, we compared obtained results from our previous experiments (Błońska et al., 2021) where as a stressor we used elevated temperature (+10°C). Similarly, we kept single individual for 24 h in the same experimental arena. Then we moved fish to identical tanks equipped with heater maintaining temperature at 28–29°C. For further comparisons we used data for males only.

Experimental protocol had three treatments, which lasted 1, 6 and 12 h (E1, E6 and E12, respectively), with six replicates of each, after which both resident and intruder fish were deprived of life by spinal cord rupture. Liver and muscle tissue were extracted and immediately frozen in temperature at −80°C for further analyses. All the experiments were carried out in accordance with Relevant guidelines and ARRIVE guidelines and the experimental study was approved by Local Ethical Committee for Animal Experiments based at the Medical University of Lodz (41/LB102/2018).

### 2.3 Biochemical analysis

Liver and muscle tissues (separately) were homogenised using a X‐120 knife homogenizer (CAT Ingenieurbüro GmbH, Germany) in 100 mM sodium phosphate buffer (pH 7.4, 100 mM KCl, 1 mM Na_2_‐EDTA) with 100 μM PMSF (Phenylmethysulfonyl fluoride) dissolved in ethanol (98%), on ice at 3500 rpm for 4 min. Then, homogenates were centrifuged at 4°C for 10 min (15,000 rpm) and supernatants were removed for further analysis. Total protein was determined based on Lowry et al. (1951) method and used to calculate results obtained for other tested parameters. For each sample, three technical replicate measurements were performed.

Antioxidant defence was evaluated based on the level of reduced glutathione (GSH) (Ellman, 1959) and the activity of catalase (CAT) (Aebi, 1984), and oxidative damage was measured using the concentration of the end products of lipid peroxidation (MDA) (Rice‐Evans et al., 1991). Additionally, total antioxidant capacity (TAC) (Bartosz, 2003) as a general activity of the antioxidant system was assessed. All methods were described and successfully used in our previous study on round goby (Błońska et al., 2021).

### 2.4 Behavioural analysis

All experimental arenas were connected to video camera infrastructure, which enabled observing and recording fish behaviour in high resolution. Regarding the experimental protocol, we considered 1, 6 and 12 h of each trial for observations. We recorded following behaviours: aggression, guarding the shelter and time spent in the shelter. Aggression was defined as attacks and chases; guarding the shelter as leaning the head out of the shelter; time spent in the shelter as staying hidden in the shelter. Aggressive behaviours and guarding the shelter were counted in each treatment and expressed as a mean number of events per hour. Time spent in the shelter was expressed as a mean time based on all observations and calculated per hour.

### 2.5 Statistical analysis

Permutational univariate analysis of variance (PERANOVA) in PERMANOVA+ v.1.0.1 for PRIMER version 6.1.11 (PRIMER‐E LTD, Plymouth, UK) was used to examine differences in oxidative stress parameters between tissue, treatment and status (resident/intruder). PERMANOVA holds an advantage over conventional parametric analysis of variance due to its relaxation of the strict assumptions of data normality and homoscedasticity. These assumptions, frequently unmet when working with ecological datasets, are notably eased (Anderson, 2001). It has robustness by testing for significance by comparing the actual *F* value to a value gained from random permutations of the objects between groups. Furthermore, it tests for similarity based on a distance measure instead of group means. It has widely been used in several previous similarly designed studies (Almeida et al., 2014; McCarthy et al., 2014; Weber & Traunspurger, 2016).

Additionally, we performed similar analysis comparing the combined results (resident and intruder) with previously obtained data on round goby males (Błońska et al., 2021), exchanging status with type of stressor evaluated (i.e. competition or temperature). The aim of this analysis was to evaluate the strength of the competition by comparing it to a strong stressor such as heat shock (especially that we used the same experimental arena, similar protocol of the experimental design and the same species). A three‐way (fully crossed) data analysis design was used with fixed factors (Tissue, Treatment and Status/Experiment). After normalisation of the data, Euclidean distance measure was used to obtain a distance matrix, which was subjected to 9999 permutations of the raw data and tested for significance, followed by a posteriori pairwise comparisons evaluated at *α* = .05.

The observed fish behaviour was analysed by log‐linear analysis (Queen et al., 2002) to determine the effect of exposure time (1/6/12 h) and fish status (resident/intruder). First, a null model (i.e. assuming equal frequencies) was fitted, adding factors sequentially, ranging from all possible combinations of single factors, up to a saturated model (including Time of exposure × Status interaction). The significance level for the subsequent analysed factors (*α* = .05) was then tested by the analysis based on the chi‐square test. Fitting of log‐linear models was performed in the R and the programming environment for statistical calculations and 64‐bit graphics ver. 2.13.0 (R Core Team, 2023), using the MASS v7.3‐58.2 library in the Poisson distribution.

## 3 RESULTS

**FIGURE 1:**
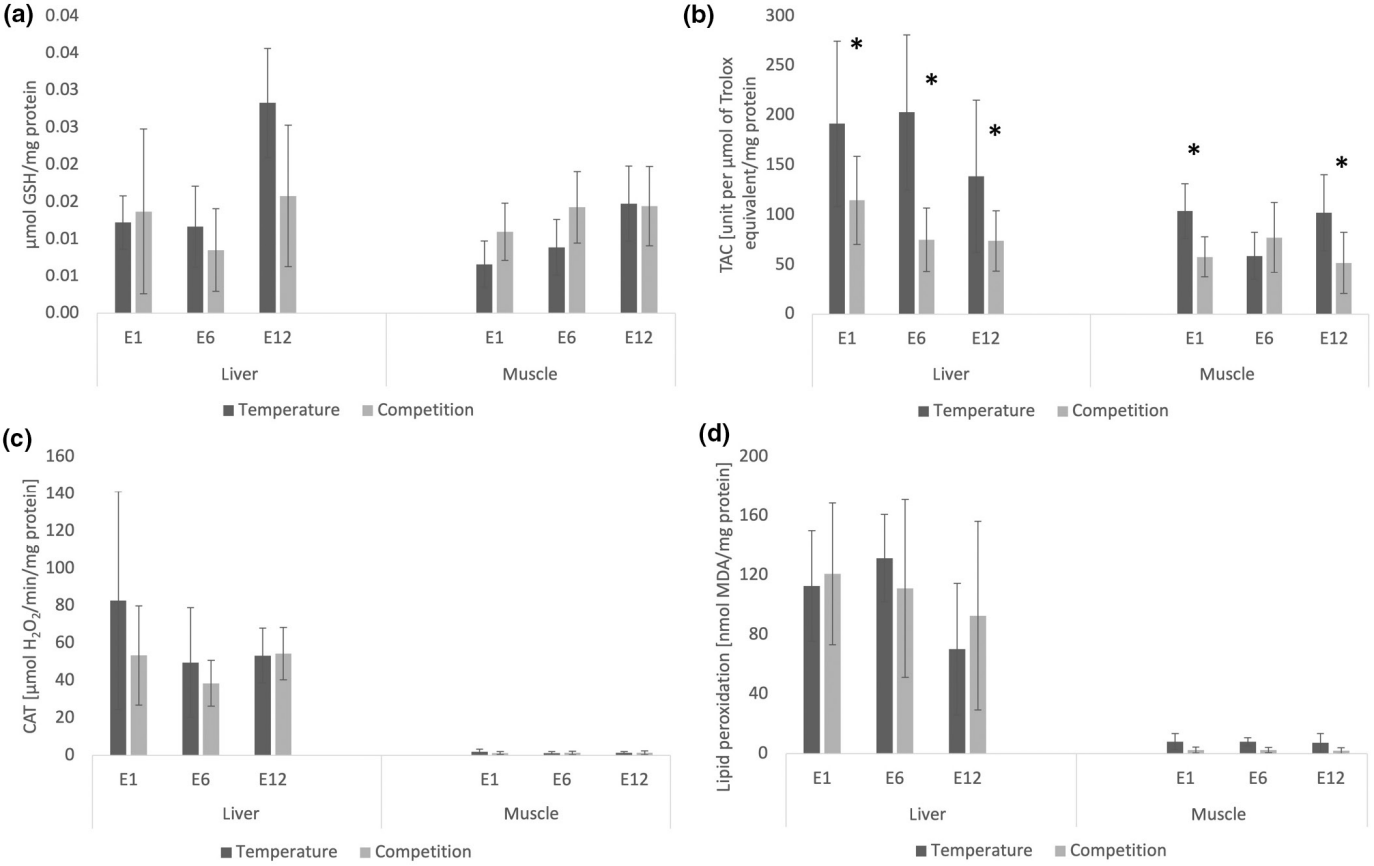
Levels of (a) reduced glutathione, (b) total antioxidant capacity, (c) catalase activity, (d) lipid peroxidation (mean ± SD) measured in tissues of round goby males exposed to elevated temperature (+10°C; from 19 to 29°C) and presence of competitor for 1, 6 and 12 h (E1, E6 and E12, respectively) (*n* = 6 for temperature and *n* = 12 for competition related to combining the resident and intruder data). Significant differences between stressors in particular tissues were marked by asterisk (*p* < .05). Results presented for elevated temperature were published in Błońska et al. (2021).

The obtained results from both experiments, expressed as the mean with standard deviation, have been illustrated in Figure 1. In the context of competition, all examined parameters exhibited negligible divergence between the resident and intruder, with scarcely discernible distinctions among the various treatment (time of exposure). The most notable variance was observed among tissues. Consequently, we decided to consolidate the presentation of the competition results, integrating both experiments into a unified representation.

### 3.1 Effect of competition on oxidative stress parameters

#### 3.1.1 GSH

Level of reduced glutathione in liver decreased after 6 h of exposure to competitor presence in liver and was consecutively increasing in muscle; however, these differences were not significant (Table 1).

**TABLE 1:** PERMANOVA results on the differences in reduced glutathione (GSH) level, total antioxidant capacity (TAC), catalase activity (CAT) and lipid peroxidation (LPO) in round goby males by status (resident/intruder), tissue (liver/muscle) and time of exposure (1, 6 and 12 h; E1, E6 and E12, respectively).

Source of variation	df	MS	*F* ^a^	*t* ^a^	*p* ^a^
GSH					
Status	1	0.0352	0.034		.8644
Treatment	2	1.6495	1.608		.2149
Tissue	1	0.1068	0.104		.7545
Status × Treatment	2	0.0510	0.050		.9509
Status × Tissue	1	0.8665	0.845		.3648
Treatment × Tissue	2	2.2933	2.235		.1131
Status × Treatment × Tissue	2	0.2268	0.221		.8082
Residual	60	1.0258			
TAC					
Status	1	1.7137	2.171		.1502
Treatment	2	2.3367	2.960		.0605
Tissue	1	8.3983	10.64		**.0009**
Status × Treatment	2	0.0143	0.018		.9833
Status × Tissue	1	1.1617	1.472		.2294
Treatment × Tissue	2	3.7592	4.763		**.0111**
E1					
Liver vs. muscle				4.049	**.0003**
E6					
Liver vs. muscle				0.159	.8731
E12					
Liver vs. muscle				1.752	.0938
Status × Treatment × Tissue	2	0.0731	0.092		.9146
Residual	60	0.7893			
CAT					
Status	1	0.8601	3.976		**.0542**
Treatment	2	0.6205	2.869		.0619
Tissue	1	53.289	246.37		**.0001**
Status × Treatment	2	0.1269	0.587		.5541
Status × Tissue	1	0.8460	3.911		.0527
Treatment × Tissue	2	0.6230	2.880		.0619
Status × Treatment × Tissue	2	0.1431	0.662		.5263
Residual	60	0.2163			
LPO					
Status	1	0.6971	2.048		.1557
Treatment	2	0.2864	0.842		.4232
Tissue	1	45.467	133.59		**.0001**
Status × Treatment	2	0.6697	1.968		.1545
Status × Tissue	1	0.7034	2.067		.1610
Treatment × Tissue	2	0.2663	0.782		.4563
Status × Treatment × Tissue	2	0.6332	1.861		.1652
Residual	60	0.3404			

*Note*: Statistically significant effects in bold (*α* = .05) including those for a posteriori pairwise comparisons.

^a^ Permutational value based on 9999 permutations.

#### 3.1.2 TAC

Significant Treatment × Tissue interaction (Table 1) indicated differences between tissues and between time of exposure to stressors. First hour of exposure was more intensively expressed in liver compared with muscle, and this value was also significantly higher than in 6‐ and 12‐h treatment.

#### 3.1.3 CAT

Significant tissue factor indicates differences between liver and muscle with higher values observed in liver tissue (Table 1).

#### 3.1.4 LPO

Significant tissue factor indicates differences between liver and muscle with higher values observed in liver tissue (Table 1).

### 3.2 Comparison between stressors: Competition versus acute heat shock

#### 3.2.1 GSH

Differences between exposition to both stressors were pronounced significantly within muscle tissue and at the threshold of significance in liver (Experiment × Tissue interaction; Table 2).

**TABLE 2:** PERMANOVA results on the differences in reduced glutathione (GSH) level, total antioxidant capacity (TAC), catalase activity (CAT) and lipid peroxidation (LPO) in round goby males by experiment (elevated temperature/competition), tissue (liver/muscle) and time of exposure (1, 6 and 12 h; E1, E6 and E12, respectively).

Source of variation	df	MS	*F* ^a^	*t* ^a^	*p* ^a^
GSH					
Experiment	1	0.2530	0.348		.5610
Treatment	2	9.9981	13.375		**.0001**
Tissue	1	4.5920	6.317		**.0134**
Experiment × Treatment	2	3.3099	4.553		**.0134**
Experiment × Tissue	1	6.2670	8.621		**.0037**
Liver					
Temperature vs. competition				2.0159	**.0492**
Muscle					
Temperature vs. competition				2.4192	**.0179**
Treatment × Tissue	2	2.7456	3.777		**.0249**
Liver					
E1 vs. E6				1.0501	.3163
E1 vs. E12				2.8065	**.0076**
E6 vs. E12				4.5661	**.0001**
Muscle					
E1 vs. E6				1.9164	.0628
E1 vs. E12				3.6351	**.0007**
E6 vs. E12				1.7504	.0930
Experiment × Treatment × Tissue	2	0.7249	0.997		.3833
Residual	96	0.7270			
TAC					
Experiment	1	22.523	43.168		**.0001**
Treatment	2	1.4391	2.758		.0639
Tissue	1	22.231	42.610		**.0001**
Experiment × Treatment	2	0.0267	0.051		.9529
Experiment × Tissue	1	6.8305	13.092		**.0003**
Treatment × Tissue	2	2.6537	2.543		.0868
Experiment × Treatment × Tissue	2	2.8962	5.551		**.0052**
E1; liver					
Temperature vs. competition				2.6025	**.0156**
E6; liver					
Temperature vs. competition				5.0129	**.0003**
E12; liver					
Temperature vs. competition				2.6166	**.0135**
E1; muscle					
Temperature vs. competition				4.0559	**.0012**
E6; muscle					
Temperature vs. competition				1.1592	.2734
E12; muscle					
Temperature vs. competition				3.0218	**.0082**
Residual	96	5217			
CAT					
Experiment	1	0.9882	2.986		.0941
Treatment	2	1.1251	3.400		**.0380**
Tissue	1	65.430	197.73		**.0001**
Experiment × Treatment	2	0.4636	1.401		.2487
Experiment × Tissue	1	0.9470	2.862		.0920
Treatment × Tissue	2	1.0546	3.187		**.0467**
Liver					
E1 vs. E6				2.1908	**.0299**
E1 vs. E12				1.3593	.1892
E6 vs. E12				1.6366	.1130
Muscle					
E1 vs. E6				1.1664	.2519
E1 vs. E12				0.8181	.4221
E6 vs. E12				0.3637	.7129
Experiment × Treatment × Tissue	2	0.4157	1.256		.2905
Residual	96	0.3310			
LPO					
Experiment	1	0.0067	0.020		.8910
Treatment	2	0.9681	2.896		.0607
Tissue	1	60.984	182.46		**.0001**
Experiment × Treatment	2	0.2368	0.709		.4894
Experiment × Tissue	1	0.1215	0.363		.5481
Treatment × Tissue	2	0.9062	2.711		.0717
Experiment × Treatment × Tissue	2	0.2315	0.693		.5000
Residual	96	0.3342			

*Note*: Statistically significant effects in bold (*α* = .05) including those for a posteriori pairwise comparisons.

^a^ Permutational value based on 9999 permutations.

#### 3.2.2 TAC

Significant Experiment × Treatment × Tissue interaction (Table 2) indicated differences in total antioxidant capacity between stressors (elevated temperature vs. competition) depending on tissue and time of exposure (treatment). Within liver and muscle tissues, round gobies responded more intensively to elevated temperature than competitor presence. Exposed to both stressors, round goby males showed higher values of TAC level in liver than in muscle in almost all treatments (Table 2). Considering liver, in each treatment elevated temperature exposition resulted in higher values of TAC, for muscle only in 6‐h treatment this difference was not significant.

#### 3.2.3 CAT

Catalase activity was higher in liver than in muscle in each treatment, with highest values after first hour of exposition (Experiment × Tissue interaction; Table 2).

#### 3.2.4 LPO

Significant tissue factor indicates differences between liver and muscle with higher values observed in liver tissue (Table 2). In muscle, elevated temperature led to higher oxidative damages, which were three times higher, compared with competitor presence, however, these differences were not significant. In liver, there were no significant differences regarding the stressor.

#### 3.2.5 Behaviour

**FIGURE 2:**
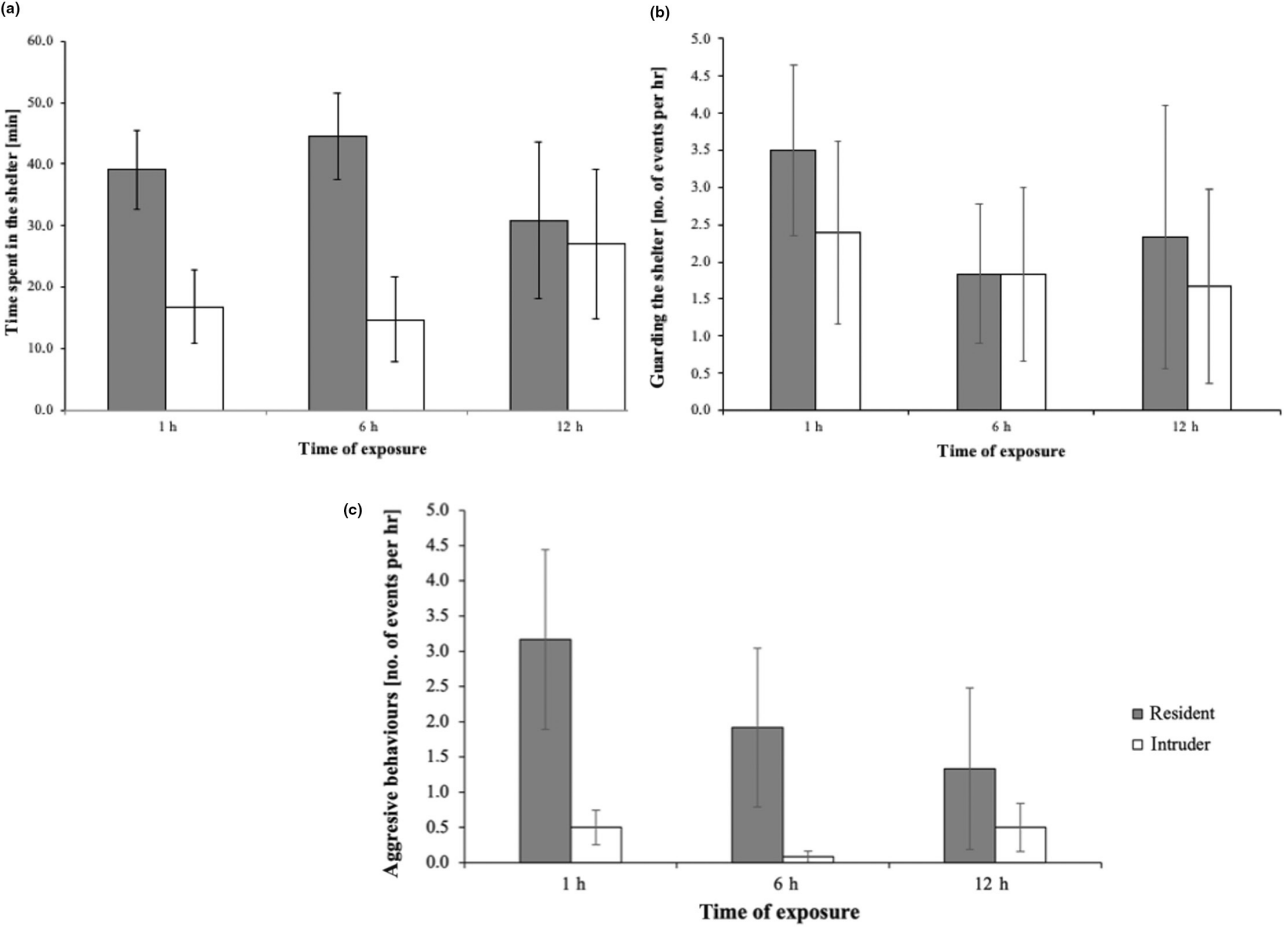
(a) Time spent in the shelter, (b) guarding the shelter, (c) aggressive behaviours (±SD) displayed by resident (dark) and intruder (white) round goby male during the 1, 6 and 12 h treatment.

Time spent in shelter depended on time of exposure and fish status (significant Time × Status interaction; Table 3). Residents spent more time staying the shelter than intruders (Figure 2a). However, shelter occupation decreased with time of exposition (1 > 6 > 12 h). This trend was also observed in the frequency of guarding the shelter (Figure 2b). Aggressive behaviours were more often displayed by the resident fish, and most intensive within the first hour (Figure 2c).

**TABLE 3:** Results of log‐linear analysis for behaviour displayed by round goby males competing for a shelter (factor status: resident/intruder) in the experiment (factor: time of exposition 1/6/12 h).

Source of variation	df	Deviance	Resid. df	Resid. dev.	Pr (>chi)
Time spent in the shelter					
Null model			71	2606.3	
Time	2	1.566	69	2604.7	.4569
Status	1	303.811	68	2300.9	**<.0001**
Time × Status	2	50.738	66	2250.2	**<.0001**
Aggressive behaviours					
Null model			71	331.97	
Time	2	9.728	69	322.25	**.0077**
Status	1	62.461	68	259.79	**<.0001**
Time × Status	2	3.773	66	256.01	.1516
Guarding the shelter					
Null model			71	432.35	
Time	2	8.4827	69	423.87	**.0144**
Status	1	3.3209	68	420.55	.0684
Time × Status	2	1.1452	66	419.40	.5641

*Note*: Significant effects (*α* = .05) were bolded.

## 4 DISCUSSION

Our results demonstrated that the presence of competitors did not elicit any significant response, and no distinctions were observed between resident and intruder fish across all tested parameters. Notably, the liver tissue exhibited heightened sensitivity, as evidenced by higher values in comparison to muscle tissue.

The appearance of intruding fish provoked the resident to active defence the shelter resource. The higher activity resulting from aggressiveness may have led to an increase in ROS production observed in increased TAC and CAT activity (however, not significant in our study), triggering a response from the antioxidant system and resulting in observed oxidative damage (Costantini, 2008; Dijkstra et al., 2011; Leeuwenburgh & Heinecke, 2001). Considering increased aerobic metabolism due to physical activity such as threats, chases and bites, muscle tissue was found to be significantly less responsive compared with the liver, and this was also observed for round goby in the present study. The resident goby spent more time in the shelter, defending the resource and was also more aggressive. Aggressive behaviours were most intensively exhibited within the 1 h of exposure.

Recent studies on social competition indicate significant association between aggression and oxidative condition (Border et al., 2019, 2021; Isaksson et al., 2011; Mentesana & Adreani, 2021). For example, in White's skink lizards *Egernia whitii* aggressive male phenotype was positively correlated with antioxidant capacity (Isaksson et al., 2011), whereas contrasting results were found in horneros *Furnarius rufus*, where acute aggressive interactions decreased the antioxidant capacity in males and even more strongly in females (Mentesana & Adreani, 2021). In our study of round goby, antioxidant defence measured via catalase activity (CAT), reduced glutathione (GSH) and total antioxidant capacity (TAC) was generally not‐status dependent and was more pronounced in liver tissue. CAT activity varied between tissues, where it dropped after 6 h of exposure in the liver and increased after 12 h, whereas in muscle tissue, it increased with the following treatments, however these differences were not significant in both tissues. The activity of this enzyme differs among tissues and is one of the most responsive (Hermes‐Lima, 2004). Catalase is most effective when the hydrogen peroxide concentration is highly elevated (Hermes‐Lima, 2004), whereas at low concentrations, glutathione peroxidase (GPx) has a higher affinity (Bartosz, 2003). GSH levels showed reversed pattern in liver and muscle (not significant), reaching their lowest and highest values in 6 h treatment (liver and muscle, respectively). One of the possible explanation of such a situation might be the support provided by liver, the main GSH supplier, due to lower levels of GSH in muscle (Błońska et al., 2021; Hermes‐Lima, 2004; Lushchak, 2012). In our study, both the resident and intruder round goby belonged to the same species, and most of oxidative stress parameters are species‐specific (Birnie‐Gauvin et al., 2017). Thus, differences in intraspecific interactions were probably less pronounced compared with possible interspecific ones.

Fish behaviour and antioxidant defence did not show any potential relationship reflecting changes in antioxidant status and aggression. Especially, there was no difference between resident and intruder fish in antioxidative parameters. However, regarding oxidative damage, there were noticeable similar trends in the level of lipid peroxidation and aggression acts, particularly in the resident goby, with highest aggression and highest oxidative damage within 1 h and subsequent temporal decrease (however, not significant). A positive correlation between oxidative damage and increased aggression resulting in territory defence was also shown in a cichlid fish, where dominant male endured higher oxidative damage than subordinate. Dijkstra et al. (2011) showed that aggression might be correlated with higher oxidative stress, especially in the social context, in males of two coexisting sibling cichlid species. Results of experiments conducted by Border et al. (2021) suggested an oxidative cost of social dominance regarding TAC measured in liver tissue under unsuitable environment (unstable communities), but the same parameter in other tissues (gonad and muscle) showed no effect of status in cichlids. Experiments conducted on various female morphs of other cichlid species (*Neochromis omnicaeruleus*) showed no differences in oxidative stress despite the metabolism and pigmentation disparity between morphs (Dijkstra et al., 2015). The hierarchical structure displayed in a population divides individuals into dominants and subordinates, with the former usually taking advantage of their state, which can also be reflected in its antioxidant system (Border et al., 2021; Cram et al., 2014; Funnell et al., 2021). Although the final status of an individual depends on the competitive advantage, in the current study, we used simple model (one‐on‐one) of acute competitive interaction for a limited resource. Likely, under natural conditions, losing the shelter will force defeated individual to seek for another place to establish a nest. Round goby can occur at a high abundance and dominate the fish assemblages, especially in its non‐native range (Erős et al., 2005; Polacik et al., 2008). This shows that despite high aggressiveness and competition, they succeed in establishing dense population and range expansion. Shelter places differ in their quality, for example safety against predators they provide or prey availability in the adjacent feeding grounds. Thus, less competitive individuals are most probably pushed to less optimal places.

Considering exposure to both stressors, acute heat shock and competition, elevated temperature was found to elicit a higher response of antioxidant defence than intraspecific interactions. Total antioxidant capacity (TAC), a general measure of antioxidant defence, and CAT in male round goby reached higher values in response to abiotic stress, although not always statistically significant. Compared with competition, oxidative damage due to heat shock was even three times greater in muscle, suggesting that thermal stress can be overriding stressor compared to behavioural interactions (Paul et al., 2021). In the present study, we compared the results of short‐term exposure to abiotic and biotic stressors, where we used heat shock as an abiotic factor with a sudden increase in temperature by 10°C (Błońska et al., 2021) versus abrupt introduction of a counterpart. Such a substantial change in water temperature must have been noticeable, especially for fish, which are ectotherms. Temperature increase influences nearly all biological processes, including steady‐state of ROS. Enhanced metabolism evoked by temperature increase is expected to induce oxidative stress (Birnie‐Gauvin et al., 2017; Lushchak, 2011); however, changes in behaviour related to greater muscular activity were shown to influence oxidative damage, especially for acute and unaccustomed physical actions (Monaghan et al., 2009). Studies on the effect of heat shock (sudden temperature elevation by 10–12°C) on freshwater fish species confirm increased oxidative damage measured by level of lipid peroxidation/TBARS (Bagnyukova et al., 2007; Heise et al., 2006; Kaur et al., 2005), which was also observed in round goby (Błońska et al., 2021). Our main aim was to evaluate the strength of competition in regarding a strong and certain stressor. Both stressors featured a sudden change in established conditions of resident fish. Abiotic stressor was found to be stronger for round goby males in certain parameters measured and resulted in higher oxidative damage, especially in muscle tissue.

Although our study found that acute heat shock was a more intense stressor compared to competition, we acknowledge that under natural conditions, round goby often interact with native species, and gobies usually have an advantage due to their aggressive behaviour (e.g. Bergstrom & Mensinger, 2009; Dubs & Corkum, 1996; Kornis et al., 2012). Therefore, the effect of interspecific competition could have a much stronger influence on oxidative stress parameters, especially for native species (Oliveira et al., 2015). Given that life‐history traits are connected with oxidant status (Monaghan et al., 2009), the result of such interspecific interactions could have an adverse impact on indigenous species.

## AUTHOR CONTRIBUTIONS

**Dagmara Błońska:** Conceptualization (equal); data curation (equal); funding acquisition (lead); investigation (equal); project administration (lead); resources (lead); supervision (equal); validation (equal); visualization (equal); writing – original draft (lead); writing – review and editing (equal). **Ali Serhan Tarkan:** Formal analysis (lead); investigation (equal); software (lead); validation (equal); writing – original draft (equal); writing – review and editing (equal). **Bartosz Janic:** Conceptualization (equal); investigation (equal); project administration (equal); supervision (supporting); validation (equal); visualization (equal); writing – original draft (supporting); writing – review and editing (equal). **Mariusz Tszydel:** Investigation (equal); project administration (equal); validation (equal); writing – review and editing (equal). **Bożena Bukowska:** Conceptualization (equal); investigation (equal); methodology (lead); supervision (equal); validation (equal); visualization (equal); writing – original draft (equal); writing – review and editing (equal).

## CONFLICT OF INTEREST STATEMENT

The authors declare no conflicts of interest.

## Data Availability

All data constituting the basis of the present study, including part of the data used from previous study, are available on https://doi.org/10.18150/EQYYO1.
